# Altered Macrophage and Dendritic Cell Response in* Mif*−/− Mice Reveals a Role of Mif for Inflammatory-Th1 Response in Type 1 Diabetes

**DOI:** 10.1155/2016/7053963

**Published:** 2016-09-06

**Authors:** Yuriko Itzel Sánchez-Zamora, Imelda Juarez-Avelar, Alicia Vazquez-Mendoza, Marcia Hiriart, Miriam Rodriguez-Sosa

**Affiliations:** ^1^Unidad de Biomedicina, Facultad de Estudios Superiores (FES) Iztacala, Universidad Nacional Autónoma de México (UNAM), 54090 Tlalnepantla, MEX, Mexico; ^2^Carrera de Optometría, FES-Iztacala, UNAM, 54090 Tlalnepantla, MEX, Mexico; ^3^Departamento de Neurodesarrollo y Fisiología, Instituto de Fisiología Celular, UNAM, 04510 Coyoacán, MEX, Mexico

## Abstract

Macrophage migration inhibitory factor (Mif) is highly expressed in type 1 diabetes mellitus (T1DM). However, there is limited information about how Mif influences the activation of macrophages (M*φ*) and dendritic cells (DC) in T1DM. To address this issue, we induced T1DM by administering multiple low doses of streptozotocin (STZ) to* Mif−/−* or wild-type (Wt) BALB/c mice. We found that* Mif−/−* mice treated with STZ (*Mif−/−*STZ) developed lower levels of hyperglycemia, inflammatory cytokines, and specific pancreatic islet antigen- (PIAg-) IgG and displayed reduced cellular infiltration into the pancreatic islets compared to Wt mice treated with STZ (WtSTZ). Moreover, M*φ* and DC from* Mif−/−*STZ displayed lower expression of MHC-II, costimulatory molecules CD80, CD86, and CD40, Toll-like receptor- (TLR-) 2, and TLR-4 than WtSTZ. These changes were associated with a reduced capacity of M*φ* and DC from* Mif−/−*STZ to induce proliferation in ovalbumin-specific T cells. All the deficiencies observed in* Mif−/−*STZ were recovered by exogenous administration of recombinant Mif. These findings suggest that Mif plays a role in the molecular mechanisms of M*φ* and DC activation and drives T cell responses involved in the pathology of T1DM. Therefore, Mif is a potential therapeutic target to reduce the pathology of T1DM.

## 1. Introduction 

T1DM is an autoimmune-mediated disease characterized by selective destruction of insulin-producing pancreatic *β*-cells, resulting in the need for lifelong administration of exogenous insulin for patient survival [1], and represents 5–10% of all cases of diabetes [2, 3]. The pathological condition begins with an autoimmune inflammatory process known as insulitis, which leads to leucocyte infiltration into the pancreatic islets of Langerhans, resulting in the autoimmune destruction of pancreatic *β*-cells [4]. Thus, the onset of T1DM is the result of the recognition of self-antigens due to molecular mimicry against infection with various viruses such as Coxsackie virus, *β*-cell cytotoxicity, or *β*-cell cytolysis [5–7]. Consequently, molecules such as preinsulin, insulin, glutamate decarboxylase-65 (GAD-65), and islet antigen 2*β* become recognized as self-antigens [8].

Different types of innate immune cells such as T cells, eosinophils, macrophages (M*φ*), and dendritic cells (DC) as well as proinflammatory cytokines and chemokines are present during insulitis [4, 9]. The relationship between T1DM and high levels of inflammatory cytokines such as interleukin- (IL-) 1*β* [10–14], interferon- (IFN-) *γ* [15], tumor necrosis factor- (TNF-) *α* [16], IL-12 [17, 18], and macrophage migration inhibitory factor (Mif) [19–24] has been widely recognized.

Mif is a pleiotropic cytokine produced during the immune response by activated T cells, M*φ*, DC, and a variety of nonimmune cells and plays a pivotal role in the systemic inflammatory immune response by promoting the production of proinflammatory cytokines including TNF-*α* and IL-6, which are involved in inflammatory and autoimmune diseases such as septic shock [25], cancer [26], inflammatory bowel disease [27, 28], rheumatoid arthritis [29, 30], obesity [31, 32], and diabetes [33–35]. Moreover, Mif has recently been proposed as a diagnostic biomarker for autoimmune diseases [36] such as arthritis [37, 38], type 2 diabetes [35], and ulcerative colitis [39] in both animals and humans.

The pathogenic contribution of Mif to T1DM was demonstrated by showing that Mif mRNA expression was upregulated in splenic lymphocytes during the development of spontaneously diabetic nonobese diabetic (NOD) mice, as well as cyclophosphamide-treated NOD mice. Diabetes incidence was increased to 86% in NOD mice treated with recombinant Mif (rMif) protein, compared with the 55% incidence observed in untreated control NOD mice [20]. Furthermore, studies performed using* Mif−/−* mice rendered diabetic by administering multiple low doses of streptozotocin (STZ) have shown that the absence of Mif affected several aspects of experimental T1DM, including initial immunopathological events and the production of proinflammatory and cytotoxic mediators, thereby interfering with both inflammation and tissue destruction [22].

All the results described above provide evidence that Mif plays a critical role in the pathogenesis of T1DM. However, the precise mechanism by which Mif promotes insulitis and the mechanism underlying its proinflammatory role remain unclear. The activities of Mif may reside at the levels of both the inductive and effector phases of the inflammatory response attributed to antigen-presenting cells.

Here, we analyzed the influence of Mif on M*φ* and DC activation using an autoimmune diabetes model in which multiple low doses of STZ were administered to* Mif−/−* and wild-type (Wt) mice (*Mif−/−*STZ and WtSTZ, resp.) in the BALB/c genetic background. As previously demonstrated* Mif−/−*STZ developed less severe hyperglycemia, reduced levels of IFN-*γ* and TNF-*α*, a smaller amount of pancreatic islet antigen- (PIAg-) specific IgG, and decreased cell infiltration into the pancreatic islets compared to WtSTZ. Interestingly, we found for the first time that M*φ* and DC from* Mif−/−*STZ displayed decreased expression of costimulatory molecules CD80, CD86, and CD40, as well as Toll-like receptor- (TLR-) 2, TLR-4, and major histocompatibility complex- (MHC-) II. Importantly, we demonstrated that due to diminished upregulation of costimulatory molecules, these cells exhibited a reduced capacity to induce proliferation and cytokine expression in cocultures with allogeneic ovalbumin- (OVA-) specific T cells. All deficiencies observed in* Mif−/−*STZ were reversed by exogenous rMif protein administration.

## 2. Materials and Methods

### 2.1. Ethics Statement

All experiments in this study were performed according to the guidelines in the Mexican Regulations on Animal Care (NOM-062-ZOO-1999, 2001) and were approved by the local Institutional Animal Care and Use Committee. All efforts were made to minimize animal suffering over the course of these studies.

### 2.2. Animals

Six- to 8-week-old male BALB/c mice were purchased from Harlan Laboratories (Mexico City, Mex.) and were maintained as a breeding colony in a pathogen-free environment at our animal facilities in accordance with institutional guidelines.* Mif−/−* mice were kindly provided by Dr. Abhay R. Satoskar (The Ohio State University, USA) and were maintained as breeding colonies for more than 10 generations in the BALB/c genetic background on the Transgenic Mouse Core Facility at our institution. Genotyping of* Mif−/−* mice was routinely performed on DNA isolated from tail snips using a PCR procedure [40]. The PCR were performed using the following primers: Mif: forward 5′-AGACCACGTGCTTAGCTGAG-3′ and reverse 5′-GCATCGCTACCGGTGGATAA-3′; Neomycin (Neo): forward 5′-ATTGAACAAGATGGATTGCAC-3′ and reverse 5′-CGTCCAGATCATCCTGATC-3′. PCR for the amplification of Mif and NEO was performed by adding 100 ng of the extracted DNA to 25 *μ*L of a reaction mixture that contained 18.4 *μ*L of distilled water, 2.5 *μ*L of 10x PCR buffer, 0.4 *μ*L of dNTPs (10 mM), 1.5 *μ*L of MgCl_2_ (25 mM), 1 *μ*L of the forward and reverse primers, and 0.2 *μ*L (2.5 units) of Taq DNA polymerase (Ampliqon, Bioreagents and Molecular Diagnostics). The amplification protocol consisted of 5 min at 95°C, 35 cycles of 95°C for 30 sec, 58°C for 40 sec, and 72°C for 30 sec and a final extension at 72°C for 5 min. All PCR experiments were conducted with positive and negative controls. A PCR fragment of 200 bp, corresponding to Mif, or 500 bp, corresponding to NEO, was visualized to identify Wt or* Mif−/−* mice, respectively. The PCR products were analyzed by electrophoresis on a 1.5% agarose gel and were viewed under UV light (Bio-Rad, USA).

### 2.3. Induction of T1DM

*Mif−/−* and Wt mice were deprived of food for 8 h before induction of diabetes via intraperitoneal (i.p.) injection of STZ at doses of 40 mg/kg of body weight, daily for five consecutive days (days 0–4) (Sigma-Aldrich, St. Louis, MO, USA). STZ was diluted in cold 0.01 M citrate buffer (pH 4.5) and was used within 5 min of preparation, in accordance with a previously reported protocol [41]. Healthy mice from each group received i.p. injections of an equivalent volume of vehicle (citrate buffer) as negative controls.

### 2.4. Analysis of Blood Glucose, Serum Insulin, and Cytokine Levels

Blood samples were collected by tail snipping from Wt and* Mif−/−* mice that had been fasting for 6 h. Samples were obtained once before STZ injection and 2, 4, and 8 weeks after STZ injection. Blood glucose levels were measured with a portable glucometer (Accu-Chek Sensor glucometer; Roche Diagnostics, Indianapolis, IN, USA). Mice with a glucose concentration exceeding 300 mg/dL were considered to have T1DM. Blood was collected and centrifuged at 1300 ×g, and the serum levels of Mif (Neobiolab, USA), IL-12, IFN-*γ*, IL-17, IL-4, IL-13 (PeproTech, Mex), and insulin (Lincoln, St. Charles, MO, USA) were determined via ELISA according to the manufacturer's instructions.

### 2.5. Pancreatic Islets Antigen (PIAg) Isolation

PIAg islets were obtained from healthy Wt mice as described below. After isolation, the islets were lysed by five freeze-thaw cycles followed by sonication (six 60-Hz cycles for 1 min each) on ice. After centrifugation (10 000 g, 15 min, 4°C), supernatants were collected and filtered through a 0.2 *μ*m membrane (Corning, Cambridge, MA, USA). The protein concentration was determined using the Lowry method [42], and PIAg aliquots were stored at −70°C until use.

### 2.6. Analysis of IgG Antibody Production

Serum samples were analyzed for the levels of pancreatic islet-specific Th1-associated IgG2a and Th2-associated IgG1 antibodies by ELISA. Briefly, 96-well ELISA plates (Costar) were coated with 100 *μ*L/well soluble PIAg in Tris buffer, pH 7.8. The plates were incubated overnight at 4°C. Then, the wells were washed thoroughly with phosphate-buffered saline (PBS) containing 0.05% Tween-20 (PBS-Tween; Merck, France) and were blocked with PBS supplemented with 1% bovine serum albumin (PBS-BSA; Sigma-Aldrich) for one hour at room temperature (RT). The serum samples were diluted 1 : 100, followed by serial dilution of each sample (in PBS-BSA) from healthy or STZ-treated Wt or* Mif−/−* mice. The plates were then incubated at 4°C overnight. After extensive washing with PBS-Tween, the samples were incubated for 45 min at RT with isotype-specific peroxidase-labeled goat anti-mouse antibodies (anti-IgG1 and anti-IgG2a at 1/1000 dilutions; Zymed, San Francisco, CA, USA). Then, the plates were washed, and immunoreactivity was detected with ABTS solution (Zymed). The results were expressed as endpoint titers based on optical density.

### 2.7. Histopathology

Pancreatic tissues from Wt and* Mif−/−* mice healthy or injected with STZ were removed, fixed overnight in 4% formaldehyde, and embedded in paraffin blocks. Afterwards, 5 to 7 *μ*m transverse sections of pancreatic tissue were sliced from the paraffinized tissue blocks, mounted on slides, and subsequently stained with eosin-hematoxylin (E&H; Sigma-Aldrich). For each mouse, one histological containing 1–3 nonsuccessive slices was scored for infiltration as previously described [43] according to the following scale: grade 0 (no insulitis) = 0% infiltration within the islets; grade 1 (peri-insulitis) = 1–10% infiltration; grade 2 (moderate insulitis) = 11–<50% infiltration; grade 3 (severe insulitis) = >50% infiltration; or grade 4 (complete insulitis) = extensive infiltration with few or no detectable pancreatic islet cells. Using an Olympus BX51 microscope (Olympus America, Melville, NY, USA) equipped with a digital video camera, 30 islets of Langerhans were evaluated per mouse.

### 2.8. Cell and Pancreatic Islet Isolation

Spleen and pancreatic islet cells from WtSTZ and* Mif−/−*STZ were collected after 0, 2, 4, and 8 weeks of injection with STZ and stained for flow cytometry analysis. Briefly, the spleen was removed under sterile conditions, and spleen cells were obtained by mincing and filtering the tissue, followed by washing and suspension in DMEM culture medium supplemented with 10% fetal bovine serum, 100 units of penicillin/streptomycin, 2 mM glutamine, and 1% nonessential amino acids (all from GIBCO, BRL, Grand Island, NY, USA). Spleen cells were suspended at 5 × 10^6^ cells/mL in the same medium. The pancreas was also removed under sterile conditions, and pancreatic islets were isolated using the collagenase method as previously described [44]. Briefly, the pancreas was removed and cut into small pieces (approximately 3 mm in size). The tissue was subsequently incubated in collagenase (0.3 mg/mL; Roche Diagnostics Corp., Indianapolis, IN, USA) for 10 minutes at 37°C in a total of 1 mL of digestion solution under constant shaking and intermittent vortexing. Islets were subsequently washed several times in HBSS containing BSA (5 mg/mL) and were hand-picked under a dissecting microscope. Islets were dispersed into single cells by suspension in trypsin-EDTA (GIBCO, BRL) and passage through a siliconized Pasteur pipette. Then, the cells were incubated at 37°C.

### 2.9. Flow Cytometry Analysis

Cells from the spleen or pancreas obtained as described above were then used for flow cytometry analysis. In brief, cells were washed in flow cytometry wash solution (Dulbecco's PBS containing 1% FCS and 0.05% sodium azide), followed by incubation with allophycocyanin- (APC-) conjugated anti-F4/80 and anti-CD11c antibodies for differentiation of M*φ* and DC, respectively. Then, the selected cells were incubated in 3% BSA-PBS containing a phycoerythrin- (PE-) labeled anti-CD80, anti-CCR5, or anti-TLR-4 antibody or a fluorescein isothiocyanate- (FITC-) labeled anti-CD86, anti-CD40, anti-MHC-II, or anti-TLR-2 antibody (all antibodies from Biolegend, San Diego, CA, USA) at 4°C for 30 min. After incubation, the cells were washed several times in buffer, fixed in 1% paraformaldehyde (Sigma-Aldrich), and stored at 4°C in the dark, followed by analysis using a FACSCalibur flow cytometer and CellQuest software (Becton Dickinson, Franklin Lakes, NJ, USA).

### 2.10. Coculture of Macrophages with Spleen Cells

Coculture of M*φ* with naive spleen cells was performed as follows. Adherent M*φ* among peritoneal exudate cells (PECs) from healthy or 8 weeks after STZ-treatment Wt or* Mif−/−* mice were obtained. Briefly, the M*φ* density was adjusted to 5 × 10^6^ cells/mL, and the cells were plated (100 *μ*L) in 96-well flat-bottom plates (Costar, Cambridge, MA, USA). Three hours later, the PECs were washed three times with warm sterile PBS to remove nonadherent cells, and 10 *μ*g of OVA (Worthington, USA) in 100 *μ*L of DMEM supplemented media was added. Three hours later, adherent M*φ* were washed three times with warm sterile DMEM to remove excess OVA that had not been phagocytosed. Spleen cells from OVA-transgenic mice were obtained as previously described [45], suspended at 1 × 10^6^ cells/mL, and added (100 *μ*L) to PECs at a ratio of 1 M*φ* : 5 spleen cells. The cocultures were maintained at 37°C in 5% CO_2_ for 5 days. Then, [3H]-thymidine (185 GBb/mmol activity, Amersham, UK) was added at 0.5 *μ*Ci/well, and the cells were incubated for a further 18 h. The cells were harvested using a 96-well harvester (Tomtec, Toku, Finland) and then counted using a microplate counter (Trilux, Toku, Finland). The values are presented as counts per minute (CPM) from triplicate wells.

### 2.11. Mif Reconstitution

To establish the Mif levels under conditions of T1DM, the serum Mif levels were determined weekly until 8 weeks after STZ treatment in WtSTZ.* Mif−/−*STZ received similar concentrations of rMif (R&D Systems, USA) to the Mif levels observed in WtSTZ to emulate physiological conditions in WtSTZ. Briefly,* Mif−/−* mice received i.p. injection of STZ together with 500 pg of rMif in 100 *μ*L of saline solution as a vehicle. Upon STZ treatment,* Mif−/−* mice received an i.p. injection of rMif every three days at the following doses: 850 pg on week 1; 1280 pg on week 2; 1292 pg on week 3; 1305 pg on week 4; 4300 pg on week 5; 7300 pg on week 6; and, finally, 13200 pg on week 7. Other* Mif−/−*STZ mice were injected with an equivalent volume of saline solution (100 *μ*L) as controls.

### 2.12. Statistical Analysis

Comparisons between Wt and* Mif−/−* mice that were healthy or treated with STZ were performed using either Student's unpaired *t*-test or ANOVA followed by Turkey's multiple comparisons test for data that displayed a normal distribution. *p* values less than 0.05 were considered significant and were designated as ^*∗*^ *p* < 0.05, ^*∗∗*^ *p* < 0.01, or ^*∗∗∗*^ *p* < 0.001. All data were analyzed using GraphPad Prism 6 software (San Diego, CA, USA).

## 3. Results

### 3.1. *Mif*−/− Mice Developed Less Severe Hyperglycemia Than Wt Mice after STZ Administration

**Figure 1:**
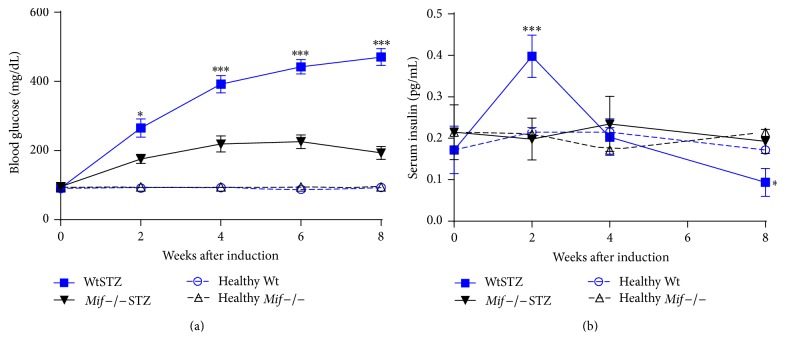
*Mif−/− *mice developed less severe hyperglycemia than Wt mice after type 1 diabetes mellitus induction. Blood glucose (every 2 weeks) (a) and serum insulin (0, 2,, and 8 weeks) (b) levels were monitored as described in Section 2 in Wt (■) and* Mif−/−* (▼) mice after STZ administration; as controls, healthy Wt (○) or healthy* Mif*−/− (Δ) mice were plotted as well. All data are representative of three independent experiments and are expressed as the means ± SE (*n* = at least 5–7 animals per group/time point). ^*∗*^ *p* < 0.05, ^*∗∗∗*^ *p* < 0.001, GraphPad Prism software 6.

We first investigated the clinical effects of STZ on* Mif−/−* and Wt mice. Our data demonstrated that Wt mice rapidly developed hyperglycemia after STZ administration. These mice sustained high blood glucose levels from week 2 (281.7 ± 16 mg/dL) until week 8 of STZ administration (470.5 ± 24 mg/dL) (Figure 1(a), WtSTZ: squares). In contrast, hyperglycemia developed gradually in* Mif−/−*STZ. The blood glucose level of* Mif−/−*STZ peaked at 219 ± 23 mg/dL on week 4, and the blood glucose level in some mice decreased between weeks 6 and 8 (226 ± 15 and 182 ± 16 mg/dL, resp.) (Figure 1(a),* Mif−/−*STZ: inverted triangles). This observation suggested that a slight recovery of the glucose response may occur in* Mif−/−*STZ at approximately week 8 of STZ treatment.

WtSTZ showed a peak blood insulin level at 2 weeks after STZ treatment, and after the sixth and eighth weeks of treatment, their blood insulin levels were significantly lower than those of healthy Wt mice (Figure 1(b), WtSTZ: squares with solid line; Wt mice: white circles with dotted line). Interestingly,* Mif−/−*STZ showed no significant changes in insulin levels compared to healthy* Mif−/−* or Wt mice over the course of the experiment (Figure 1(b),* Mif*−/−STZ: inverted triangles with solid line; healthy* Mif−/−* mice: white triangles with dotted line).

### 3.2. *Mif*−/− Mice Produced Lower Proinflammatory Cytokine Levels Than Wt Mice after T1DM Induction

**Figure 2:**
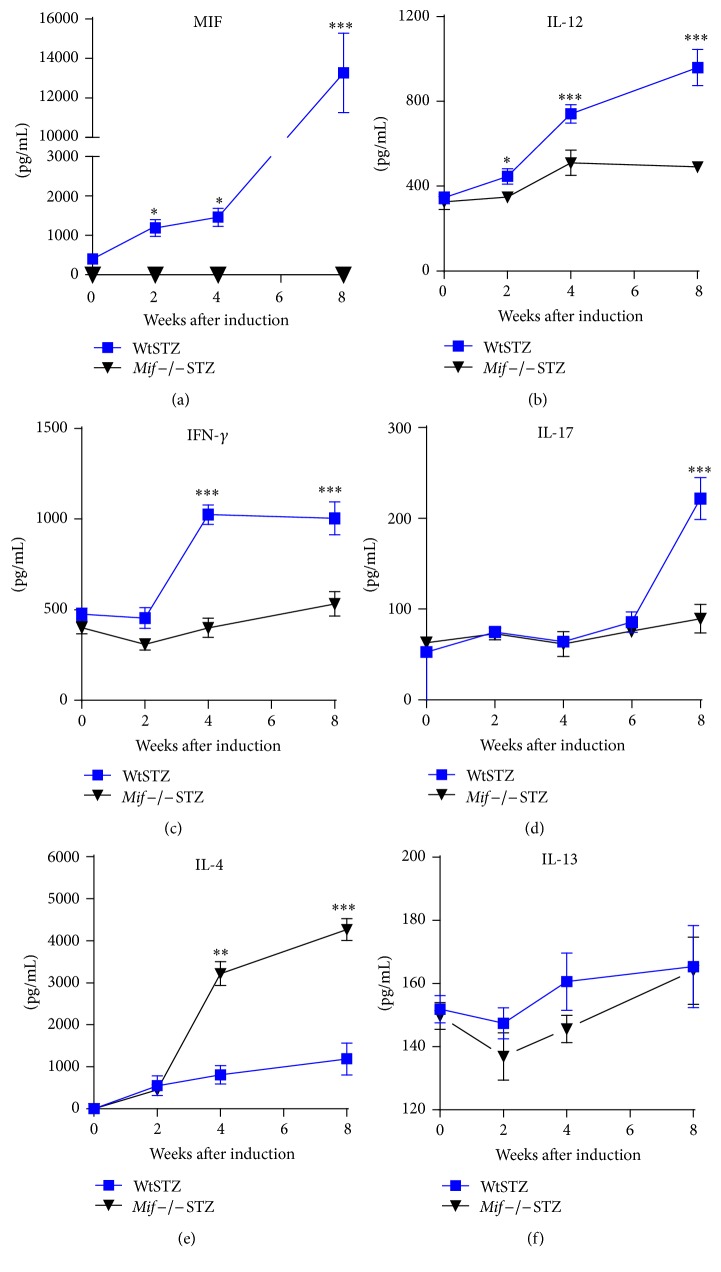
*Mif *deficiency prevents the elevation of proinflammatory cytokine production. The levels of Mif (a), IL-12 (b), IFN-*γ* (c), IL-17 (d), IL-4 (e), and IL-13 (f) in the sera from Wt (■) and* Mif*−/− (▼) mice at 0, 2, 4, and 8 weeks after STZ administration were measured via enzyme-linked immunosorbent assay (ELISA) in triplicate, as indicated in Section 2. Data are expressed as the means ± SE (*n* = at least 5–7 mice per group/time point). ^*∗*^ *p* < 0.05, ^*∗∗*^ *p* < 0.01, or ^*∗∗∗*^ *p* < 0.001, GraphPad Prism software 6.

We measured the serum levels of proinflammatory and anti-inflammatory cytokines in Wt and* Mif−/−* mice after STZ administration. As expected, the WtSTZ displayed gradually increasing serum levels of inflammatory cytokines such as Mif, IL-12, and IFN-*γ* (Figures 2(a), 2(b), and 2(c), resp., WtSTZ: squares) between 2 and 8 weeks after T1DM induction. The level of the proinflammatory cytokine IL-17 was significantly increased at 8 weeks after T1DM induction in WtSTZ (Figure 2(d), squares). In contrast,* Mif−/−*STZ displayed significantly lower serum levels of Mif, IL-12, IFN-*γ*, and IL-17 than WtSTZ (Figures 2(a), 2(b), 2(c), and 2(d), resp.;* Mif−/−*STZ: inverted triangles).

The serum levels of the anti-inflammatory cytokine IL-4 were significantly lower in WtSTZ (squares) than in* Mif−/−*STZ (inverted triangles) (Figure 2(e)). No differences in the serum IL-13 levels were found between WtSTZ and* Mif−/−*STZ (Figure 2(f)).

### 3.3. *Mif*−/− Mice Showed Reduced Pancreatic Islet Damage and Cellular Infiltration Compared to Wt Mice after T1DM Induction

To confirm that STZ reached its target, Wt and* Mif−/−* mice were treated with a single high dose of STZ (150 mg/kg). The toxic effect of high-dose STZ was similar between Wt and* Mif−/−* mice (Supplementary Figure 1 in Supplementary Material available online at http://dx.doi.org/10.1155/2016/7053963). In both experimental groups, the toxic effect of STZ was present, and the high dose of STZ destroys the insulin-producers beta cells, leading to acute nonimmune-mediated diabetes; in contrast the multiple low doses of STZ at 40 mg/Kg, daily for five consecutive days, require participation of immune-inflammatory events for T1DM development [41].

To assess the damage to *β* cells in a model of T1DM induced by multiple low doses of STZ, we sacrificed the mice at 8 weeks after T1DM induction and removed the pancreas for H&E staining and histological analysis. We assessed the number and size of normal and infiltrated pancreatic islets in each slide for the experimental and healthy mice. Thirty pancreatic islets were quantified per experimental group.

**Figure 3:**
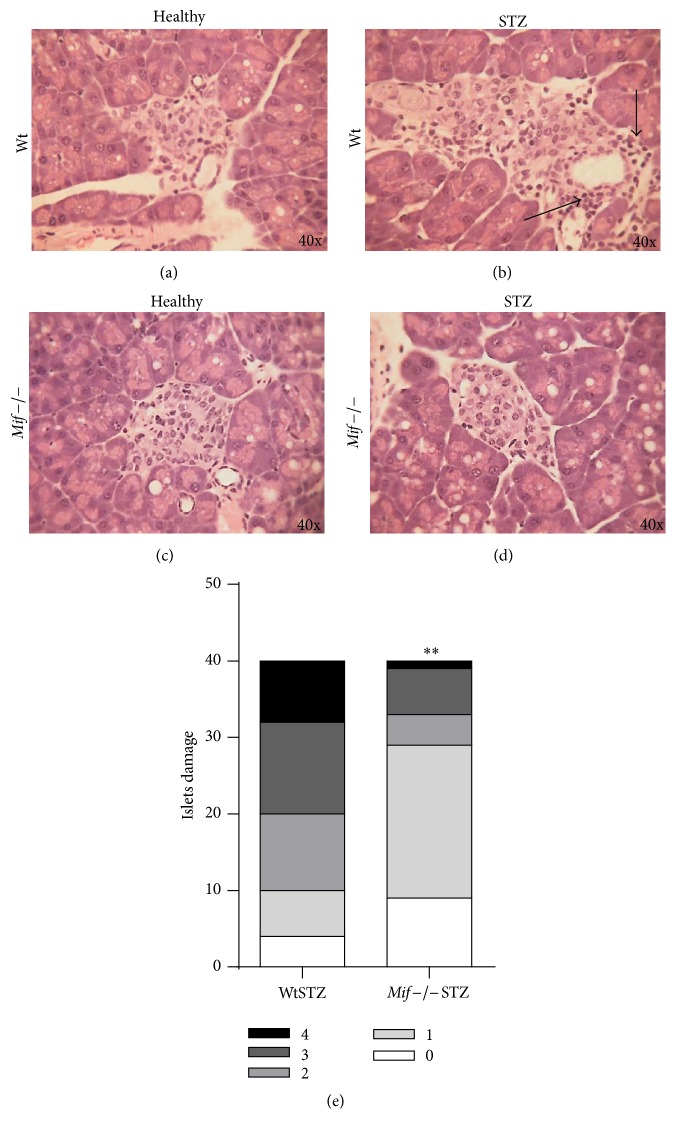
*Mif−/− *mice conserve their healthy anatomy and exhibit limited cellular infiltration into pancreatic islets after STZ administration. Pancreases isolated from Wt or* Mif*−/− mice were examined via histological analysis of eosin-hematoxylin (E&H) staining to establish the number of pancreatic islets and to determine lymphocyte infiltration (arrowhead in (b)) at 8 weeks after STZ administration. Pancreatic islets from healthy Wt mice (a); Wt mice treated with STZ, WtSTZ (b); healthy* Mif*−/− mice (c), and* Mif*−/− mice treated with STZ,* Mif*−/−STZ (d). Compilation of infiltration stages in the pancreas of Wt and* Mif*−/− mice after STZ administration (e). Pancreatic islets were scored using the following scale: grade 0 (no insulitis) = 0% infiltration; grade 1 (peri-insulitis) = 1–10% infiltration; grade 2 (moderate insulitis) = 11–<50% infiltration; grade 3 (severe insulitis) = >50% infiltration; or grade 4 (complete insulitis) = complete infiltration. We counted 30–40 islets per experiment using six mice per experimental group, depending on the number of islets that were present in the sections. All data are representative of two independent experiments, *n* = 12 from two experiments. ^*∗∗*^ *p* < 0.01, GraphPad Prism software 6.

Islets from healthy Wt and* Mif−/−* mice had a round morphology and well-defined borders without cellular infiltration (Figures 3(a) and 3(c)). As expected, the pancreas from WtSTZ showed fewer and smaller islets than those from healthy Wt mice. Additionally, evident cellular infiltration led to the breakdown of islet morphology in the pancreas of WtSTZ (Figure 3(b)). In contrast, partial islet damage (Figure 3(d)) and no significant reduction in islet number or size were observed in* Mif−/−*STZ compared to WtSTZ (Table 1).

As shown in Figure 3(e),* Mif−/−* mice at 8 weeks after STZ administration displayed marked reductions in both invasive insulitis (insulitis grades 3 and 4) and mild peri-insulitis (insulitis grade 2) in pancreatic islets compared to WtSTZ. Thus, pancreatic islets from* Mif−/−*STZ had reduced damage associated with reduced leucocyte infiltration compared to pancreatic islets from WtSTZ. These results demonstrated that STZ administration triggers *β*-cell destruction followed by insulin deficiency and hyperglycemia in Wt mice. Although STZ reached the pancreatic islets in* Mif−/−* mice,* Mif−/−*STZ exhibited minor damage compared to WtSTZ.

### 3.4. *Mif*−/− Mice Showed Lower Levels of Specific Pancreatic Islet Antibodies Than Wt Mice after T1DM Induction

In T1DM, the immune system targets self-antigens within pancreatic islets and destroys the inhabiting insulin-secreting *β*-cells. Therefore, autoantibody detection serves as a predictive factor for the onset of diabetes in both humans and mice [46, 47].

**Figure 4:**
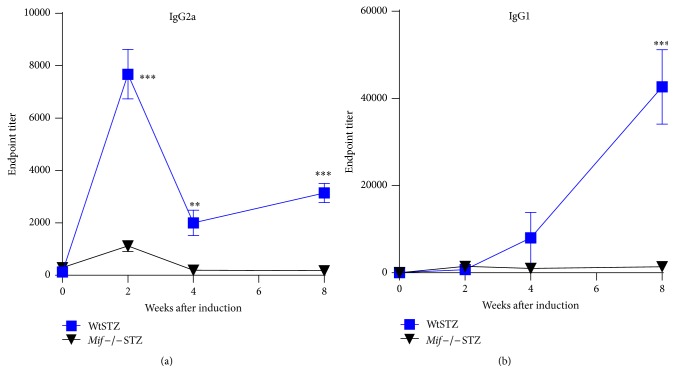
Kinetics of antibody responses in type 1 diabetes mellitus models induced by low-dose STZ. Specific production of IgG2a (a) and IgG1 (b) antipancreatic islet antigen antibodies. Sera from Wt (■) and* Mif−/−* mice (▼) at weeks 0, 2, 4, and 8 after STZ administration were processed by ELISA, using pancreatic islets antigen (PIAg) as the source of specific antigen as indicated in Section 2. Data are expressed as the mean reciprocal endpoint titer ± SEM. Five to six animals were analyzed in each group. The data shown are representative of one out of three identical experiments with similar results. Mann-Whitney* U* test. ^*∗∗*^ *p* < 0.01, ^*∗∗∗*^ *p* < 0.001, GraphPad Prism software 6.

To investigate how Mif contributes to autoantibody production in T1DM, the serum levels of pancreatic islet-specific IgG2a and IgG1 antibodies were determined in Wt and* Mif−/−* mice after 2, 4, and 8 weeks after STZ administration. WtSTZ produced high levels of IgG2a at all time points analyzed (Figure 4(a)) and IgG1 only at week 8 (Figure 4(b)). Importantly,* Mif−/−*STZ mice displayed significantly lower levels of both IgG2a and IgG1 than WtSTZ (Figures 4(a) and 4(b), resp.), suggesting suppressed development of an adaptive immune response. These findings suggest that Mif modulates the incidence and severity of diabetes by favoring the development of autoantibodies in T1DM.

### 3.5. Splenic M*φ* and DC from* Mif*−/−STZ Mice Expressed Lower Levels of Costimulatory Molecules Than Those from WtSTZ Mice

We showed that the reduced severity of T1DM in* Mif*−/−STZ is associated with decreased pancreatic islet damage and diminished adaptive immune responses. To determine whether this phenotype could be related to the degree of activation of antigen-presenting cells, we characterized the expression of the costimulatory molecules CD80, CD86, and MHC-II and of the receptors TLR-2 and TLR-4 in M*φ* and DC from the spleen and pancreas of* Mif−/−* and Wt mice after 0, 2, 4, and 8 weeks of STZ administration.

**Figure 5:**
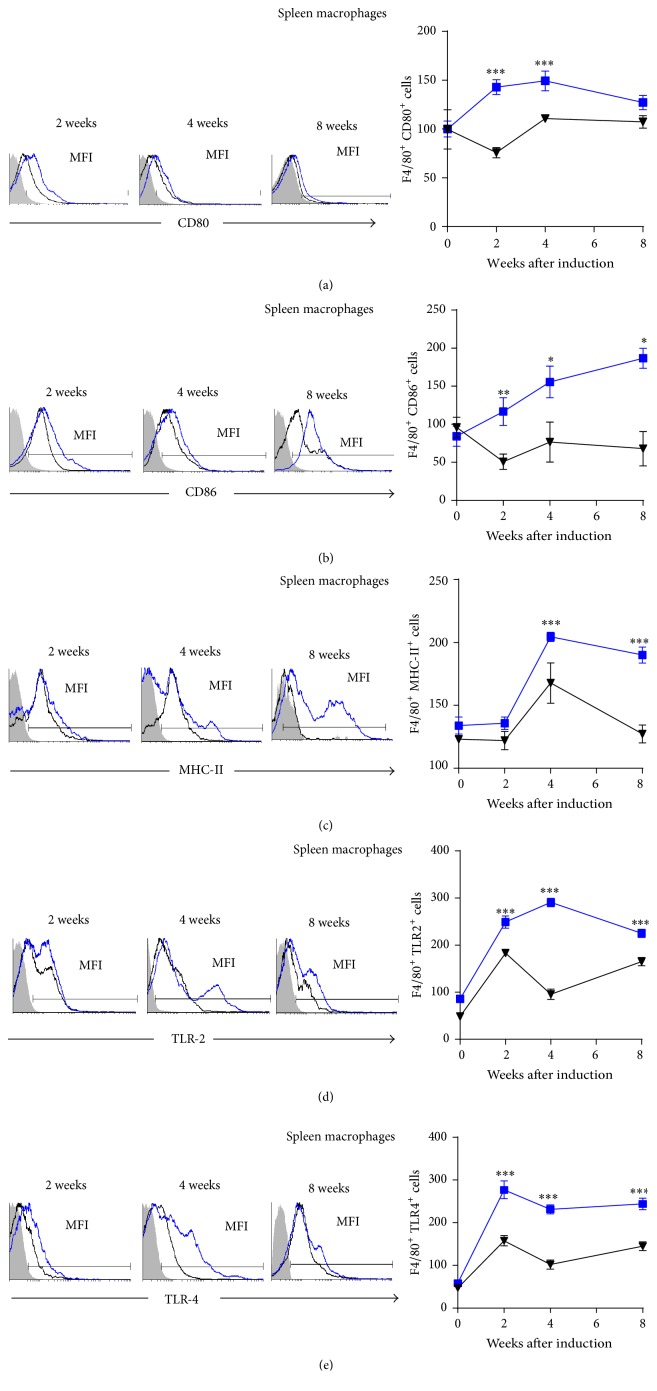
*Mif *promotes costimulatory molecule expression on spleen M*φ*. The time course of costimulatory molecule: CD80 (a), CD86 (b), MHC-II (c), TLR-2 (d), and TLR-4 (e) expression on spleen F4/80^+^ M*φ*. M*φ* from spleen of Wt and* Mif−/−* mice were harvested at 0, 2, 4, and 8 weeks after STZ administration and 1 × 10^7^ cells/mL were processed by flow cytometric analysis as indicated in Section 2. Analyses of expression are shown in the right panels: WtSTZ (■);* Mif−/−*STZ (▼). And representative histograms of expression based on fluorescence are shown in the left panels; isotype controls are indicated in gray shadow, the black line represents expression by* Mif−/−*STZ cells, and the blue line shows expression by WtSTZ cells. The data are presented as the mean fluorescence intensities (MFI) from one representative experiment. Each experiment was repeated three times (*n* = 3) and individually analyzed. ^*∗*^ *p* < 0.05, ^*∗∗*^ *p* < 0.01, or ^*∗∗∗*^ *p* < 0.001, GraphPad Prism software 6.

In cells isolated from the spleen, the percentages of CD80-, CD86-, MHC-II-, TLR2-, and TLR4-expressing M*φ* (F4/80^+^) were similar between healthy* Mif−/−* and Wt mice (Figures 5(a), 5(b), 5(c), 5(d), and 5(e), resp., at time 0 post-STZ administration, right panel). Interestingly, upon STZ administration,* Mif*−/− mouse M*φ* showed impaired activation, characterized by reduced expression of CD80, CD86, MHC-II, TLR-2, and TLR-4, compared with M*φ* from WtSTZ mice (Figures 5(a), 5(b), 5(c), 5(d), and 5(e), resp.).

**Figure 6:**
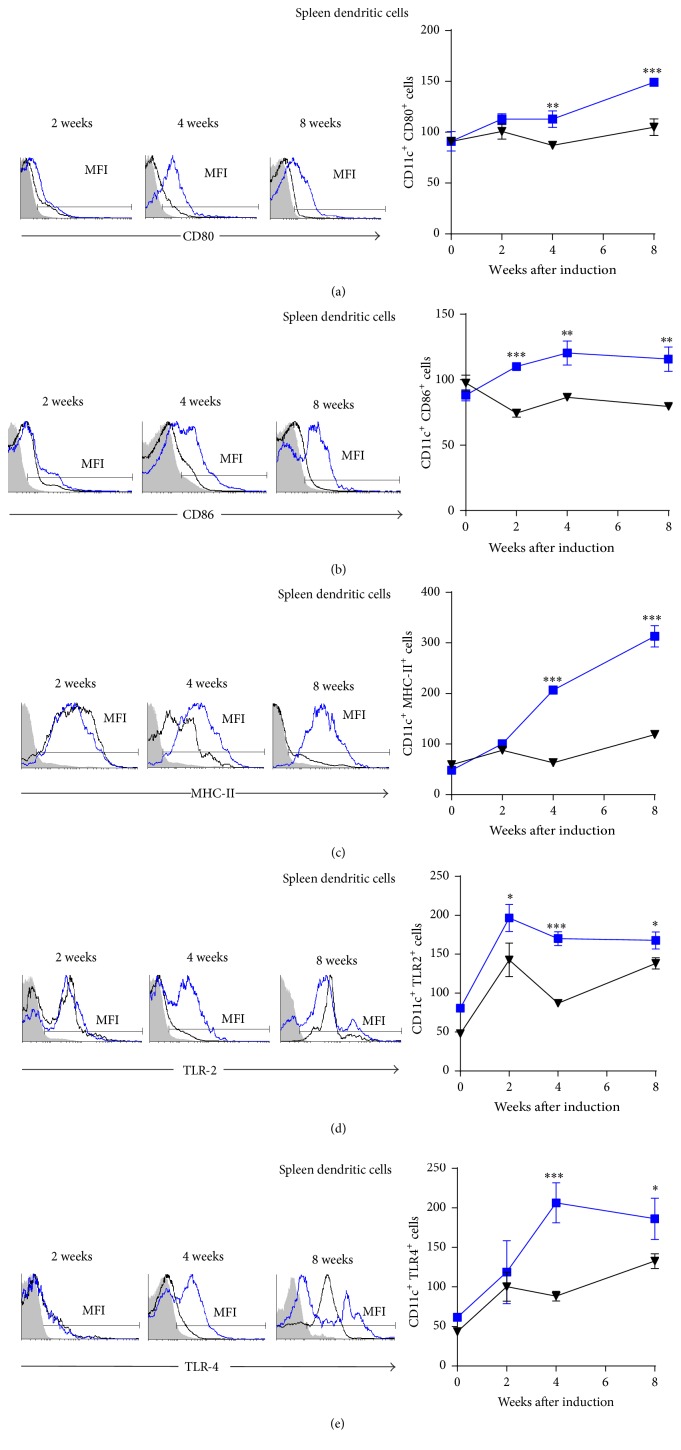
*Mif *promotes costimulatory molecules expression on spleen DC. The time course of costimulatory molecule: CD80 (a), CD86 (b), MHC-II (c), TLR-2 (d), and TLR-4 (e) expression on spleen CD11c^+^ DC. DC from spleen of Wt and* Mif−/−* mice were harvested at 0, 2, 4, and 8 weeks after STZ administration and 1 × 10^7^ cells/mL were processed by flow cytometric analysis as indicated in Section 2. Analyses of expression are shown in the right panels: WtSTZ (■);* Mif−/−*STZ (▼). And representative histograms of expression based on fluorescence are shown in the left panels. Isotype controls are indicated in gray shadow, the black line represents expression by* Mif−/−*STZ cells, and the blue line shows expression by WtSTZ cells. The data are presented as the mean fluorescence intensities (MFI) from one representative experiment. Each experiment was repeated three times (*n* = 3) and individually analyzed. ^*∗*^ *p* < 0.05, ^*∗∗*^ *p* < 0.01, or ^*∗∗∗*^ *p* < 0.001, GraphPad Prism software 6.

Similarly, DCs (CD11c^+^) isolated from the spleen of* Mif−/−*STZ mice expressed lower levels of CD80, CD86, and MHC-II at 4 through 8 weeks after STZ administration (Figures 6(a), 6(b), and 6(c), resp.), whereas the expression of TLR-2 and TLR-4 in DCs was reduced at 4 weeks in* Mif−/−*STZ compared to WtSTZ (Figures 6(d) and 6(e), resp.).

### 3.6. Pancreatic M*φ* and DC from* Mif*−/−STZ Mice Express Lower Levels of Costimulatory Molecules Than Those from WtSTZ Mice

**Figure 7:**
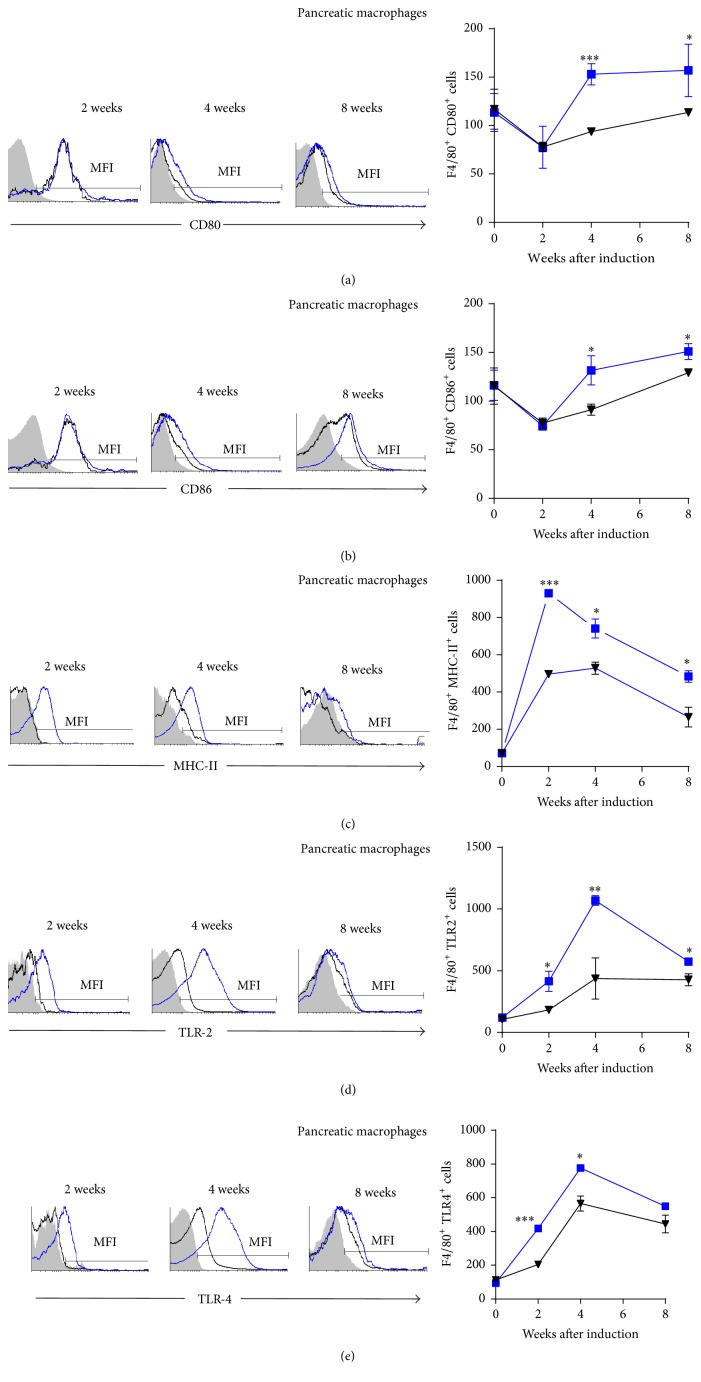
*Mif *promotes costimulatory molecule expression on pancreatic M*φ*. The time course of costimulatory molecule: CD80 (a), CD86 (b), MHC-II (c), TLR-2 (d), and TLR-4 (e) expression on pancreatic F4/80^+^ M*φ*. M*φ* from pancreas of Wt and* Mif*−/− mice were harvested at 0, 2, 4, and 8 weeks after STZ administration and 1 × 10^7^ cells/mL were processed by flow cytometric analysis as indicated in Section 2. Analyses of expression are shown in the right panels: WtSTZ (■);* Mif*−/−STZ (▼). And representative histograms of expression based on fluorescence are shown in the left panels. Isotype controls are indicated in gray shadow, the black line represents expression by* Mif−/−*STZ cells, and the blue line shows expression by WtSTZ cells. The data are presented as the mean fluorescence intensities (MFI) from one representative experiment. Each experiment was repeated three times (*n* = 3) and individually analyzed. ^*∗*^ *p* < 0.05, ^*∗∗*^ *p* < 0.01, or ^*∗∗∗*^ *p* < 0.001, GraphPad Prism software 6.

To investigate the role of Mif in the activation of APCs in the pancreas, we determined MHC-II and costimulatory molecule expression in M*φ* and DC in the pancreas of* Mif−/−*STZ and WtSTZ mice. M*φ* from* Mif−/−*STZ expressed lower levels of CD80 and CD86 from 4 through 8 weeks after STZ administration (Figures 7(a) and 7(b), resp.). Alternatively, reduced expression of MHC-II, TLR-2, and TLR-4 was detected earlier, from 2 weeks until 8 weeks after STZ treatment (Figures 7(c), 7(d), and 7(e), resp.).

**Figure 8:**
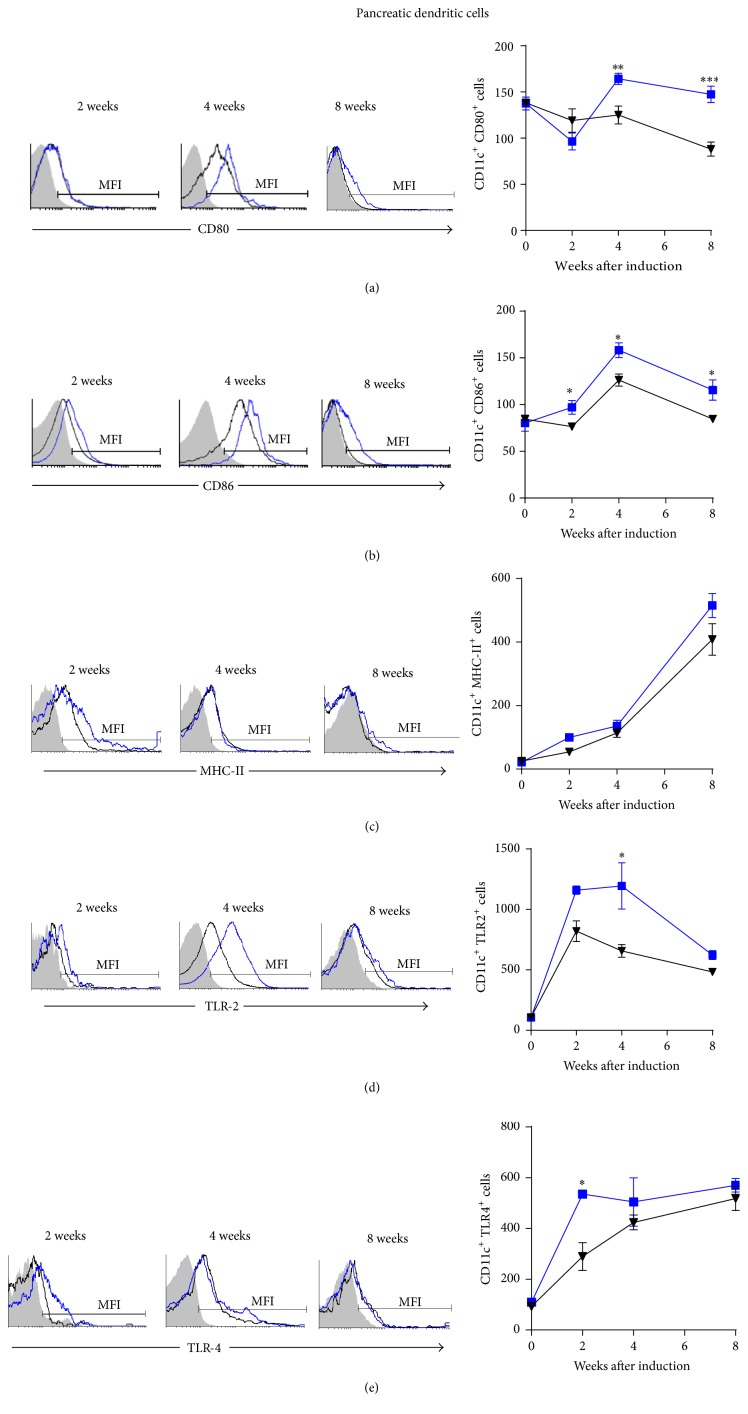
*Mif *promotes costimulatory molecules expression on pancreatic DC. The time course of costimulatory molecule: CD80 (a), CD86 (b), MHC-II (c), TLR-2 (d), and TLR-4 (e) expression on pancreatic CD11c DC. DC from pancreas of Wt and* Mif*−/− mice were harvested at 0, 2, 4, and 8 weeks after STZ administration and 1 × 10^7^ cells/mL were processed by flow cytometric analysis as indicated in Section 2. Analyses of expression are shown in the right panels: WtSTZ (■);* Mif−/−*STZ (▼). And representative histograms of expression based on fluorescence are shown in the left panels. Isotype controls are indicated in gray shadow, the black line represents expression by* Mif−/−*STZ cells, and the blue line shows expression by WtSTZ cells. The data are presented as the mean fluorescence intensities (MFI) from one representative experiment. Each experiment was repeated three times (*n* = 3) and individually analyzed. ^*∗*^ *p* < 0.05, ^*∗∗*^ *p* < 0.01, or ^*∗∗∗*^ *p* < 0.001, GraphPad Prism software 6.

Moreover, DC from* Mif−/−*STZ mice displayed reduced CD80 at 4 to 8 weeks (Figure 8(a)), CD86 at all time points analyzed (Figure 8(b)), and reduced TLR-2 and TLR-4 expression at 4 weeks and 2 weeks, respectively, compared to DCs from WtSTZ (Figures 8(d) and 8(e), resp.). No significant differences in MHC-II expression were detected (Figure 8(c)).

### 3.7. F4/80^+^ Macrophages and CD11b^+^ Monocytes Obtained from* Mif*−/−STZ Show Impaired Capacity to Induce Spleen Cell Proliferation

**Figure 9:**
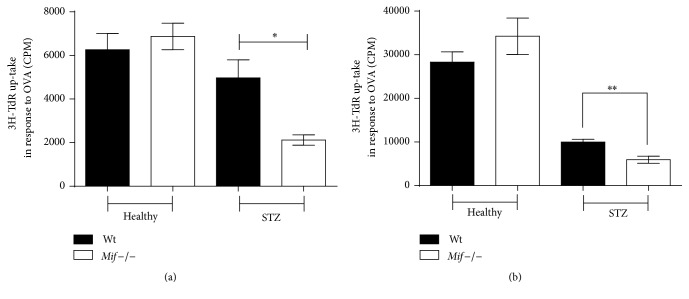
Macrophages and monocytes from* Mif−/− *mice have impaired ability to induce lymphocyte proliferation. At 8 weeks after STZ treatment, Wt (■) or* Mif−/−* (□) M*φ* (F4/80^+^) and monocytes (CD11b^+^) primed with 10 *μ*g/mL OVA were cocultured with OVA-transgenic T cells for 5 days. Subsequently, [3H]-thymidine was added for 18 h, and [3H]-thymidine incorporation was measured. The values are presented as means ± SEM counts per minute (CPM) from triplicate wells of three independent experiments (*n* = 8). ^*∗*^ *p* < 0.05 or ^*∗∗*^ *p* < 0.01. GraphPad Prism software 6, GraphPad Prism software 6.

The ability of M*φ* to activate T cells was investigated using OVA-transgenic T cell cocultures. M*φ* were collected from Wt or* Mif−/−* mice treated or untreated for 8 weeks with STZ. As shown in Figure 9(a), after priming the cells with OVA* in vitro*, M*φ* from* Mif−/−*STZ mice induced less T cell proliferation in response to OVA than M*φ* from WtSTZ. A similar trend was observed in cocultures of CD11b^+^ cells from* Mif*−/−STZ and OVA-transgenic T cells (Figure 9(b)). These data suggest a role of Mif in promoting M*φ* activation, which in turn induces specific T cell proliferation, particularly in this experimental T1DM model.

### 3.8. Mif Reconstitution in STZ-Treated* Mif*−/− Mice Promotes Hyperglycemia and Reestablishes the Production of Proinflammatory Cytokines in This T1DM Model

**Figure 10:**
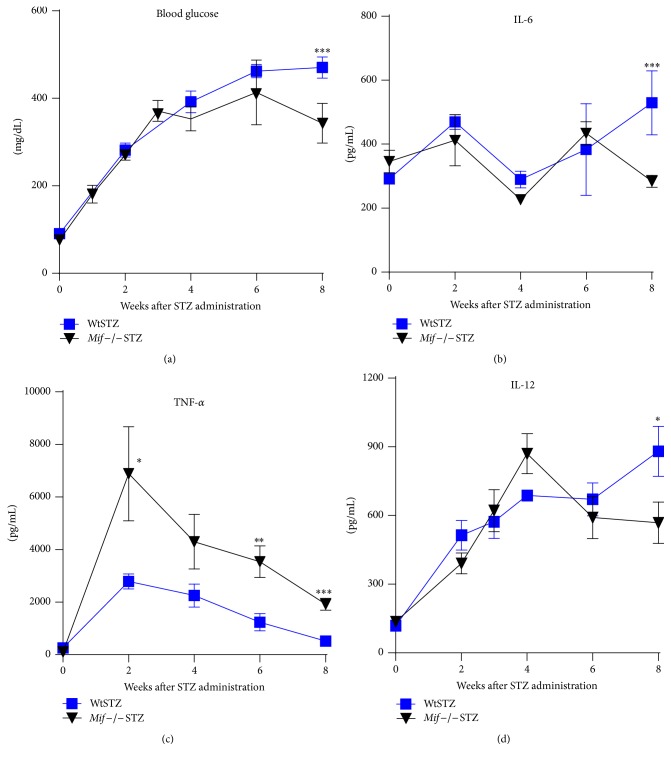
After* Mif *reconstitution, proinflammatory cytokine production in* Mif−/− *mice was increased.Mif reconstitution was initiated at the same time of STZ administration to Wt (■) or* Mif−/−* mice (▼) until 7 weeks after STZ treatment. The glucose levels (a) and the levels of the inflammatory cytokines IL-6 (b), TNF-a (c), and IL-12 (d) were measured. Control mice receiving vehicle injection did not display significant changes on cytokine production compared to week 0 (data do not shown). The values are expressed as the means ± SEM of three independent experiments (*n* = 8). ^*∗*^ *p* < 0.05, ^*∗∗*^ *p* < 0.01, or ^*∗∗∗*^ *p* < 0.001, GraphPad Prism software.

The systemic levels of Mif were reconstituted in* Mif−/−*STZ mice during the course of T1DM, as described in Section 2.* Mif−/−*STZ that received rMif (*Mif−/−*STZ+rMif) displayed blood glucose levels similar to those in WtSTZ during the first six weeks after STZ treatment. However, at 8 weeks after STZ treatment, the glucose levels were not increased in* Mif−/−*STZ+rMif compared to WtSTZ (Figure 10(a)).

Comparable serum levels of the cytokines IL-6 and IL-12 were observed between* Mif−/−*STZ+rMif and WtSTZ during the first 6 weeks after STZ treatment. However, the levels of these inflammatory cytokines significantly decreased in* Mif*−/−STZ+rMif mice at 8 weeks after STZ treatment compared to WtSTZ (Figures 10(b) and 10(d)). Interestingly, the serum levels of TNF-*α* from* Mif−/−*STZ+rMif were higher than those of WtSTZ at all time points analyzed (Figure 10(c)). These results confirm that Mif acts as a powerful inducer of proinflammatory cytokines involved in the development of experimental T1DM.

## 4. Discussion 

Currently, there is no doubt that Mif is a key molecule that promotes proinflammatory immune responses [48]. This proinflammatory property of Mif contributes to developing protective inflammatory-Th1 immune response in different models of parasitic diseases [49]. In contrast the same proinflammatory property of Mif participates in the pathogenesis of many inflammatory diseases [50]. In line with the last one, recently it has been established that high blood levels of Mif are associated with human T1DM, similar to the findings in experimental mouse models of T1DM [20, 21, 24, 51].

Studies in NOD mice or mice treated with multiple low doses of STZ (despite the pathogenic differences between these models) have shown that pancreatic *β* cell destruction results from the toxic effect of free radicals (O_2_^−^, H_2_O_2_, and nitric oxide) and inflammatory cytokines released by activated M*φ* and T cells [15, 41, 52]. Therefore, both models have been widely used to dissect the role of Mif in the pathogenesis of T1DM using anti-Mif monoclonal antibody treatment or using* Mif*−/− mice. In all cases, the lack of Mif resulted in diminished manifestation of the disease, decreased glucose blood levels, and reduced production inflammatory cytokines associated with the development of T1DM, including TNF-*α*, IL-1*β*, IFN-*γ*, IL-12, and IL-23 [21, 22, 24]. Our study validates and extends these findings by demonstrating an important role for Mif in promoting costimulatory molecule expression in M*φ* and DC during T1DM development. We further demonstrate that, in addition to regulating M*φ* and DC activation in T1DM, M*φ* isolated from T1DM* Mif−/−* mice exhibit reduced T cell activation.

Here, we observed that Wt mice treated with STZ exhibited high blood glucose levels greater than 400 mg/dL but* Mif−/−*STZ mice developed lower glucose levels of approximately 200 mg/dL, in association with lower serum levels of proinflammatory cytokines. After reconstituting Mif using exogenous rMif,* Mif−/−*STZ showed blood glucose levels and IL-6 and IL-12 levels similar to those in WtSTZ from 2 to 6 weeks after STZ treatment. Interestingly, TNF-*α* serum levels from* Mif−/−*STZ+rMif were higher than those of WtSTZ at all time points analyzed. The reduction levels of blood glucose, IL-6, and IL-12 observed in* Mif−/−*STZ mice, on week 8, probably were because the animals did not receive rMif injections on week 8, as mentioned in Section 2, so the residual effect of rMif from week 1 to week 7 was insufficient to induce IL-6 and IL-12 levels at 8 week similar to that observed in the WtSTZ mice in this point, at least for these two cytokines. These observations confirm that Mif exogenous acts as a powerful inducer of IL-6 and IL-12, but only if it is present in steady high concentration.

In addition, we found that* Mif−/−*STZ did not produce detectable levels of islet autoantibodies, in contrast to WtSTZ, which produced high levels of islet autoantibodies. It is well known that the early presence of islet autoantibodies is decisive in the development of diabetes by NOD mice as well as humans [46, 47, 53]. Our results confirm that Mif is essential for the development of hyperglycemia and suggest a role for Mif not only in the innate immune response but also in the adaptive immune response in T1DM.

Recently, it has been reported that Mif is produced by pancreatic *β*-cells and that Mif is released by insulin granules in an autocrine fashion [54, 55]. The chemical destruction of *β*-pancreatic islets by STZ in Wt mice damaged *β*-pancreatic islets; this condition could reduce one major source of Mif in this experimental T1DM model. However, we did not observe a reduction in Mif levels in this model; in contrast, Wt mice produced high serum levels of Mif after STZ administration. In line with this finding, it is known that the pancreatic islets remaining after treatment with STZ produce high levels of Mif [21] and that an elevation of Mif secretion precedes pancreatic islet death induced by IFN-*γ*, TNF-*α*, and IL-1*β* [24]. This evidence establishes that STZ did not influence Mif production/release by pancreatic islets or other cellular sources, such as T cells, DC, and M*φ* infiltrating the pancreas.

The loss of insulin production in T1DM is related to pancreatic *β*-cell destruction due to insulitis [56]. We observed that WtSTZ developed high serum levels of Mif and low insulin levels compared to* Mif−/−*STZ, which expressed insulin levels comparable to those in healthy mice. The histological analysis of pancreatic islets showed that WtSTZ displayed 100% insulitis, compared to the 33% insulitis observed in* Mif*−/−STZ mice. These observations confirm that Mif deficiency resulted in pancreatic islet protection, probably by controlling the functional activity and modulating the secretory capacity of proinflammatory cytokines produced by T cells, DC, and M*φ* that reach the pancreatic cells.

Mif has been recognized as a molecule that not only promotes proinflammatory cytokine production but also acts as a chemokine. For example, Mif plays a crucial role in leukocyte recruitment and arrest during atherosclerosis development [57]. Therefore, Mif could participate in the process of insulitis to promote the production of proinflammatory cytokines, but Mif could also promote leukocyte recruitment to pancreatic *β*-cells. For this reason,* Mif*−/−STZ exhibited reduced insulitis.

Antigen-presenting cells, M*φ* and DC, are key mediators of the development of T1DM [4]. Moreover, it has been proposed that DC orchestrate the autoimmune response in T1DM via TLR-2 and TLR-4 [58]. Previous studies by us and others have shown that Mif induces the expression of costimulatory molecules on M*φ* and DC in some pathological infections [59–61]. Here, we identified the expression of costimulatory molecules and TLR-2 and TLR-4 on M*φ* and DC, as well as the ability of M*φ* to activate T lymphocytes.

Our results demonstrated that both M*φ* and DC from the spleen and pancreas of* Mif−/−*STZ mice expressed lower levels of CD80, CD86, MHC-II, TLR-2, and TLR-4 than those of WtSTZ. These results demonstrate a role of Mif in the activation of M*φ* and DC to promote costimulatory molecule expression, which might drive pancreas-specific T cell activation and effector Th1 subset differentiation, processes that have been associated with subsequent pancreatic injury in T1DM.

In line with the results described above, we observed that both M*φ* (F4/80) and monocytes (CD11b^+^) from WtSTZ were more reactive and had greater ability to induce T lymphocyte-specific proliferation in response to OVA than those from* Mif−/−*STZ mice. This finding is consistent with the evidence that proliferation of antigen-specific T cells from TCR-transgenic mice is highly dependent on CD28/CD86 costimulation. Disrupting this interaction dramatically reduces cell proliferation [62]. This conclusion agrees with the result that blocking CD86 prevents the development of diabetes in NOD mice [63]. Moreover, mutations in the MHC-II molecule lead to development of autoimmune diabetes [64].

By another hand, the differentiation state of M*φ* is an important determinant for T cell response in T1DM. Two major populations have been defined, the classically activated (CA) M*φ* secreting proinflammatory cytokines such as TNF-*α*, IL-6, and IL-1*β* and reactive oxygen species; and alternatively activated (AA) M*φ* which secrete anti-inflammatory factors including TGF-*β* and IL-10. In T1DM it has been established that CAM*φ* trigger inflammatory responses which initiates insulitis and pancreatic *β* cell death, whereas the AAM*φ* decreases hyperglycemia, insulitis, and inflammation in the pancreas [65].

As Mif is a regulator of many proinflammatory cytokines that are characteristic for the CAM*φ*, Mif has been proposed as CAM*φ* macrophage-polarizing factor [66]. However, there are few and contrary experimental evidences about how Mif might participate in the polarization to AAM*φ* or CAM*φ*. In a mouse model of nonalcoholic fatty liver disease M*φ* in liver from* Mif−/−* mice were skewed toward AAM*φ* [67]. By contrary, in the melanoma mouse model M*φ*-derived Mif participates in AAM*φ* polarization [68]. Moreover, murine Mif and filarial nematode parasite (Brugia) Mif protein induced proinflammatory cytokines release. However, Mif also induce upregulation of IL-4 on bone marrow-derived mouse M*φ*, which when treated* in vitro* with Mif and IL-4 induce AAM*φ* [69]. Here, we show that M*φ* from* Mif−/−*STZ mice display reduced proinflammatory cytokine production and exhibit reduced ability to induce T lymphocyte proliferation in response to OVA. It is possible that Mif deficiency influences on AAM*φ* polarization in this model; however, more experiments are necessary to establish this.

## 5. Conclusions

We show for the first time a role of Mif in promoting costimulatory molecule expression in M*φ* and DC in T1DM. These results reveal Mif as a key regulator of proinflammatory function and M*φ* and DC activation in T1DM. Although more specific experiments are required, there is no doubt that Mif represents a potential target for anti-Mif therapy, which might attenuate the autoimmune process in T1DM.

## Supplementary Material

Supplementary figure: Mif deletion did not influence the non-immune "toxic" form of T1DM induced by a single high dose (150 mg/kg) of
STZ. The blood glucose levels in Wt and Mif-/- mice reached similar glucose levels. Blood glucose were determined before and every two weeks after STZ administration.

## Figures and Tables

**Table 1:** Assessment of the extent of leukocyte infiltration into pancreatic islets of WT and *Mif*−*/*− mice at 8 weeks after STZ administration.

Groups	Number of islets	Infiltrated islets	Islets that lost circular morphology
Wt	100	ND	ND
WtSTZ	100	100	65
*Mif*−/−	100	ND	ND
*Mif*−/−STZ	100	33	25

ND: none detected; Wt: wild-type.
