# Supplementary material for: Altered Macrophage and Dendritic Cell Response in Mif−/− Mice Reveals a Role of Mif for Inflammatory-Th1 Response in Type 1 Diabetes
Source: J Diabetes Res. 2016 Sep 6;2016:7053963. doi: 10.1155/2016/7053963 (PMC5028830; doi:10.1155/2016/7053963)
Supplement: Supplementary file 1 — Supplementary figure: Mif deletion did not influence the non-immune "toxic" form of T1DM induced by a single high dose (150 mg/kg) of STZ. The blood glucose levels in Wt and Mif-/- mice reached similar glucose levels. Blood glucose were determined before and every two weeks after STZ administration. [file 7053963.f1.docx]

**Supplementary figure**

Supplementary figure 1. MIF deletion did not influence the non-immune “toxic” form of T1DM induced by a single high dose (150 mg/kg) of STZ. The blood glucose levels in Wt and *Mif-/-* mice were determined before and every two weeks after STZ administration. The data are presented as the means ± SE from 10 animals per group.
